# Making (anti-) sense out of huntingtin levels in Huntington disease

**DOI:** 10.1186/s13024-015-0018-7

**Published:** 2015-04-28

**Authors:** Melvin M Evers, Menno H Schut, Barry A Pepers, Melek Atalar, Martine J van Belzen, Richard LM Faull, Raymund AC Roos, Willeke MC van Roon-Mom

**Affiliations:** Department of Human Genetics, Leiden University Medical Center, Albinusdreef 2, Leiden, 2333 ZA the Netherlands; Galapagos B.V., Leiden, the Netherlands; Department of Clinical Genetics, Leiden University Medical Center, Leiden, the Netherlands; Centre for Brain Research and Department of Anatomy with Radiology, University of Auckland, Auckland, New Zealand; Department of Neurology, Leiden University Medical Center, Leiden, the Netherlands

**Keywords:** Huntington disease, Huntingtin, Huntingtin antisense transcript, Post-mortem HD brain tissue, HD patient-derived fibroblasts, Juvenile HD

## Abstract

**Background:**

Huntington disease (HD) is an autosomal dominant neurodegenerative disorder, characterized by motor, psychiatric and cognitive symptoms. HD is caused by a CAG repeat expansion in the first exon of the *HTT* gene, resulting in an expanded polyglutamine tract at the N-terminus of the huntingtin protein. Typical disease onset is around mid-life (adult-onset HD) whereas onset below 21 years is classified as juvenile HD. While much research has been done on the underlying HD disease mechanisms, little is known about regulation and expression levels of huntingtin RNA and protein.

**Results:**

In this study we used 15 human post-mortem HD brain samples to investigate the expression of wild-type and mutant huntingtin mRNA and protein. In adult-onset HD brain samples, there was a small but significantly lower expression of mutant huntingtin mRNA compared to wild-type huntingtin mRNA, while wild-type and mutant huntingtin protein expression levels did not differ significantly. Juvenile HD subjects did show a lower expression of mutant huntingtin protein compared to wild-type huntingtin protein. Our results in HD brain and fibroblasts suggest that protein aggregation does not affect levels of huntingtin RNA and protein. Additionally, we did not find any evidence for a reduced expression of huntingtin antisense in fibroblasts derived from a homozygous HD patient.

**Conclusions:**

We found small differences in allelic huntingtin mRNA levels in adult-onset HD brain, with significantly lower mutant huntingtin mRNA levels. Wild-type and mutant huntingtin protein were not significantly different in adult-onset HD brain samples. Conversely, in juvenile HD brain samples mutant huntingtin protein levels were lower compared with wild-type huntingtin, showing subtle differences between juvenile HD and adult-onset HD. Since most HD model systems harbor juvenile repeat expansions, our results suggest caution with the interpretation of huntingtin mRNA and protein studies using HD cell and animal models with such long repeats. Furthermore, our huntingtin antisense results in homozygous HD cells do not support reduced huntingtin antisense expression due to an expanded CAG repeat.

## Background

Huntington disease (HD) is an autosomal dominant neurodegenerative disorder, characterized by motor, psychiatric and cognitive symptoms [1]. HD is caused by a CAG repeat expansion in the first exon of the *HTT* gene on chromosome 4p16, resulting in an expanded polyglutamine (polyQ) tract at the N-terminus of the huntingtin (htt) protein. People carrying 40 or more CAG repeats will develop HD, whereas people with 36 to 39 repeats show reduced penetrance [2,3]. The mean disease onset lies between 30 and 50 years of age (adult-onset HD). HD patients carrying more than 50 CAGs will have a disease onset typically below 21 years of age (juvenile HD) [1]. The major neuropathology in HD occurs in the striatum and cerebral cortex but degeneration is seen throughout the brain as the disease progresses [4] and insoluble protein aggregates in the nucleus and cytoplasm of cells are a hallmark of the disease [5].

Knowledge on regulation of HTT RNA and htt protein expression is limited and inconsistent. In patient-derived lymphoblasts, no CAG repeat-related effect on total HTT mRNA was observed [6], suggesting that there is no difference in wild-type and mutant HTT RNA expression. On the other hand, upregulation of mutant HTT mRNA translation in HD was suggested by interaction of the expanded CAG repeat with the MID1-PP2A complex [7]. Upregulation of mutant HTT mRNA translation was also suggested by HTT antisense transcript regulation [8]. Two natural HTT antisense transcripts (HTTAS) were identified at the *HTT* locus, of which one HTTAS contains a CTG repeat. Overexpression of HTTAS resulted in reduced HTT transcript levels, whereas knockdown increased HTT transcript levels [8]. Furthermore, in post-mortem HD brain no HTTAS with an expanded CTG repeat could be detected. From these observations, it was suggested that HTTAS negatively regulated HTT transcript expression and that loss of HTTAS in HD increases mutant HTT mRNA levels [8]. Upregulation of mutant HTT mRNA in human post-mortem HD brain tissue was confirmed recently using an allele-specific quantitative (q)PCR [9]. However, endogenous mRNA expression levels have not been related to subsequent mutant and wild-type htt protein levels. Also the effect of the presence of insoluble protein aggregates on htt protein levels is not known.

In this study we have examined HTT mRNA and htt protein levels in HD patient-derived fibroblasts and post-mortem brain tissue with varying CAG repeat lengths. We find a decrease in mutant HTT mRNA levels compared to wild-type in adult-onset HD patients. However, this reduced mutant HTT mRNA expression did not result in lower mutant htt protein levels. In contrast, juvenile HD fibroblasts and brain tissue did show lower levels of mutant htt protein compared to wild-type htt protein, indicating subtle differences in htt protein expression between adult-onset and juvenile HD.

## Results

### Validation of RT-PCR amplification across the CAG repeat

**Figure 1:**
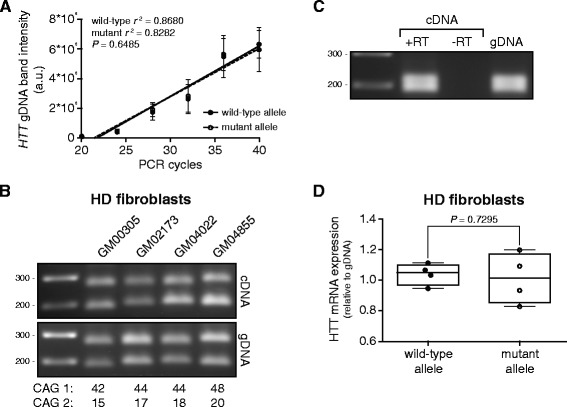
Validating RT-PCR amplification across the CAG repeat in HD patient-derived fibroblasts. Wild-type and mutant HTT were separated by gel electrophoresis. **(A)** Standard curve of wild-type and mutant HTT RT-PCR products with increasing PCR cycles from gDNA derived from post-mortem brain tissue of 7 HD patients. PCR linearity was evaluated by determining the individual linear regression coefficients (*r* ^*2*^) of the band intensities of wild-type and mutant HTT expression versus the number of PCR cycles, n = 7. **(B)** PCR products from cDNA of 4 HD (GM00305, GM02173, GM04022, GM04855) fibroblasts. CAG repeat sizes for the wild-type (lower band) and mutant alleles (upper band) are indicated below each lane. gDNA was used to examine differences in PCR amplification between the wild-type and mutant product due to the CAG repeat expansion. **(C)** RT-PCR products with input: cDNA (+RT), cDNA lacking reverse transcriptase (−RT) and gDNA of one control (GM04204). **(D)** Whisker boxplot of RT-PCR from HD patient-derived fibroblasts, comparing wild-type and mutant HTT mRNA expression levels, relative to gDNA. Line = mean, pair wise differences were evaluated using linear mixed model, n = 4.

To reliably measure both wild-type and mutant HTT mRNA we first optimized our PCR procedure to account for differences in amplification across the CAG repeat expansion. To exclude amplification bias across the CAG repeat in our PCR [10], we fitted a linear regression curve of both wild-type and mutant *HTT* genomic DNA (gDNA) with increasing PCR cycles (Figure 1A). PCR linearity was evaluated by determining the linear regression coefficient (*r*^*2*^) of the band intensities versus the number of PCR cycles. Both wild-type and mutant PCR products showed a linear increase in gel electrophoresis band intensity with increasing PCR cycle number (wild-type, *r*^*2*^ = 0.8680; mutant, *r*^*2*^ = 0.8282). The slopes were not significantly different (*P* = 0.6485), indicating that the PCR was equally efficient for both wild-type and mutant *HTT*. Additionally, we always used gDNA as a reference since gDNA has a 1:1 ratio of normal and expanded *HTT*. Next, we performed RT-PCR expansion across the CAG repeat using four adult-onset HD patient-derived fibroblasts (Figure 1B). Reverse transcription without reverse transcription enzyme was taken along, verifying that there was no gDNA contamination in our RT-PCR (Figure 1C). For each fibroblast line the two PCR products corresponding to wild-type and mutant HTT mRNA were quantified and average expression levels of wild-type and mutant HTT mRNA calculated (Figure 1D). No significant difference (*P* = 0.7642) between wild-type and mutant HTT mRNA expression was observed in adult-onset HD patient-derived fibroblasts.

### Less mutant HTT mRNA in human post-mortem HD brain material

**Figure 2:**
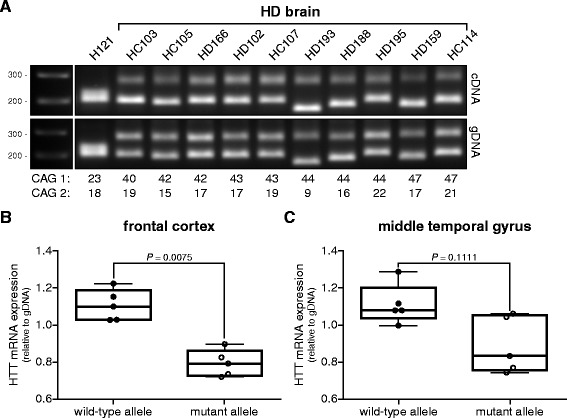
Wild-type and mutant HTT mRNA levels in adult-onset HD brain tissue. Wild-type and mutant HTT mRNA PCR products were separated on gel electrophoresis by differences in their CAG repeat length. **(A)** RT-PCR products from brain tissue derived from a control (H121) and 10 HD patients. Allelic CAG repeat sizes are indicated below each lane. gDNA from each sample was taken along to control for differences in PCR amplification efficiencies across the CAG repeat. **(B)** Whisker boxplot of allelic HTT mRNA expression levels in frontal cortex from 5 HD adult-onset HD patients. **(C)** Whisker boxplot of allelic HTT mRNA expression levels in middle temporal gyrus from 5 HD adult-onset HD patients. Expression levels relative to gDNA. Pair wise differences were evaluated using linear mixed model, n = 5.

**Figure 3:**
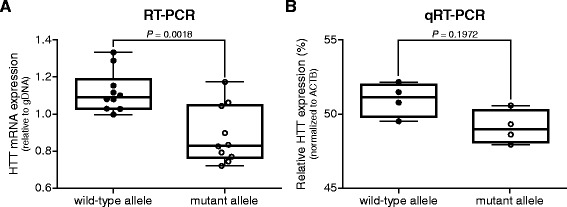
HTT mRNA quantification after RT-PCR amplification across the CAG repeat compared to SNP-specific quantitative RT-PCR. Whisker boxplots of wild-type versus mutant HTT mRNA expression levels in adult-onset HD post-mortem brain material. **(A)** Quantification after amplification across the CAG repeat, relative to gDNA. Pair wise differences were evaluated using linear mixed model, n = 10. **(B)** SNP rs362273-specific quantitative RT-PCR, normalized to β-actin (ACTB). Data were evaluated using a two-tailed student *t*-test, n = 4.

Next, we investigated HTT mRNA expression levels in post-mortem brain tissue from HD patients with a wide range of repeat lengths. RNA and gDNA was isolated from frontal cortex or middle temporal gyrus and PCR was performed with primers flanking the CAG repeat (Figure 2A). The wild-type and mutant PCR products for each brain sample were quantified and normalized against PCR products from gDNA and individual wild-type versus mutant HTT mRNA expression levels were calculated. Next, the average expression levels of wild-type and mutant HTT mRNA in the frontal cortex (Figure 2B) and middle temporal gyrus (Figure 2C) of adult-onset HD patients were calculated. We found a significant 31.0% (SEM ± 6.1%) lower average mutant HTT mRNA compared to wild-type HTT mRNA expression in the frontal cortex from HD patients. In the middle temporal gyrus, a 22.1% (SEM ± 10.9%) lower average mutant HTT mRNA expression was found. When we combined all the brain samples and repeated the analysis we found a significant lower average mutant HTT mRNA expression of 26.6% (SEM ± 6.1%) compared to wild-type HTT mRNA (Figure 3A).

To validate our results with a different technique, we performed a single nucleotide polymorphism (SNP)-specific TaqMan qPCR, using probes for rs362273 SNP located at on the 3′ side of HTT in exon 57. Of our post-mortem brain samples, 6 out of 14 were heterozygous for rs362273. Next, SNP linkage by circularization (SLiC) [11] was performed to determine which allele has the guanine and which allele the adenine in exon 57. Due to the variable RNA quality of brain tissue, SLiC was only possible in 4 out of 6 samples. TaqMan qPCR showed an identical trend towards more wild-type HTT as was found for our RT-PCR analysis (Figure 3B), but due to the smaller number of brain samples did not reach significance, demonstrating that the RT-PCR quantification across the CAG repeat is an accurate technique to measure small allelic differences in mRNA expression.

**Figure 4:**
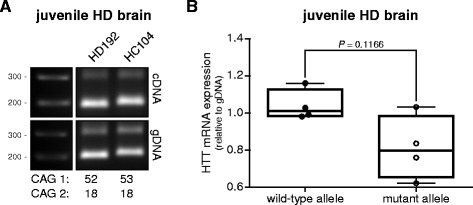
Wild-type and mutant HTT mRNA levels in juvenile HD brain. Wild-type and mutant HTT mRNA PCR products were separated by gel electrophoresis. **(A)** RT-PCR products from brain tissue derived from two juvenile HD patients (HD192 and HC104). CAG repeat sizes for the wild-type and mutant alleles are indicated below each lane. gDNA was used to control for differences in PCR amplification between the wild-type and mutant product due to the CAG repeat expansion. **(B)** Whisker box plot comparing wild-type and mutant HTT mRNA expression levels, relative to gDNA. Pair wise differences were evaluated using linear mixed model, n = 4.

Despite a lower RNA quality in our juvenile HD samples compared to the adult-onset HD samples, we were able to separate both alleles by amplification across the CAG repeat in cDNA and gDNA samples (Figure 4A). Consistent with adult-onset HD samples, we found a 22.6% (SEM ± 10.6%) lower mutant HTT mRNA expression compared to wild-type HTT mRNA in post-mortem brain tissue from juvenile HD patients (Figure 4B).

### No difference in wild-type and mutant htt protein levels in adult-onset HD

**Figure 5:**
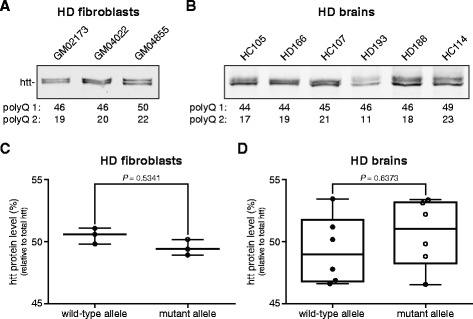
Wild-type and mutant htt protein levels in HD fibroblasts and brain tissue. **(A)** Western blot analysis of total protein lysates from human fibroblasts from three HD patients (GM02173, GM04022, GM04855). The lower band represents wild-type htt, the upper band mutant htt. PolyQ repeat lengths are indicated below each lane. **(B)** Western blot analysis of cortical brain homogenates from six HD subjects. **(C)** Whisker box plots comparing wild-type and mutant htt levels normalized against total htt in HD fibroblasts (n = 3) and **(D)** post-mortem brain tissue (n = 6). Pair wise differences were evaluated using linear mixed model.

To relate the observed differences in allelic HTT mRNA expression to wild-type and mutant htt protein levels, we analyzed SDS-soluble htt protein levels in both HD fibroblasts (Figure 5A) and post-mortem human HD brain homogenates (Figure 5B) using Western blot. Wild-type and mutant htt protein bands were quantified and both the individual wild-type and mutant htt protein levels were calculated as well as the average of all individual measurements. No significant difference between wild-type and mutant htt protein levels in patient-derived fibroblasts was found (Figure 5C). Likewise, in the post-mortem human HD brain homogenates there was no difference in wild-type and mutant htt protein levels (Figure 5D).

It is known that in post-mortem human HD brain, mutant htt aggregates in a polyQ length-dependent manner [12], while no mutant htt aggregates are present in HD patient-derived fibroblasts [13]. Our results show that the levels of soluble wild-type and mutant htt protein do not change in the absence or presence of htt protein aggregates.

### Less mutant than wild-type htt protein in juvenile HD

**Figure 6:**
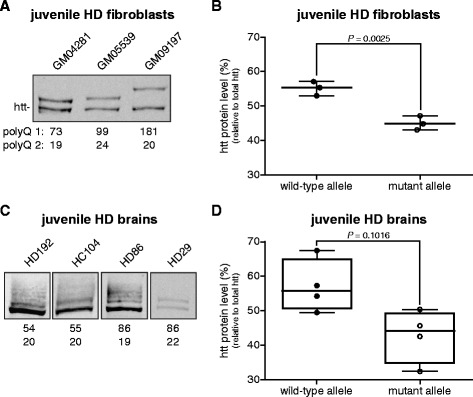
Wild-type and mutant htt protein levels in juvenile HD fibroblasts and brain tissue. **(A)** Western blot analysis of total protein lysates from human fibroblasts derived from three juvenile HD subjects (GM04281, GM05539, GM09197). The lower band represents wild-type htt, the upper band mutant htt. PolyQ repeat lengths are indicated below each lane. **(B)** Whisker box plot comparing wild-type and mutant htt levels normalized against total htt in juvenile HD fibroblasts (n = 3). **(C)** Post-mortem cortical brain tissue from four juvenile HD subjects (HD192, HC104, HD86, HD29). **(D)** Whisker box plot comparing wild-type and mutant htt levels normalized against total htt in juvenile HD post-mortem brain tissue (n = 4). Pair wise differences were evaluated using linear mixed model.

Next, we analyzed SDS-soluble wild-type and mutant htt levels in juvenile HD samples. Htt protein levels from patient-derived fibroblasts with mutant polyQ lengths of 73, 99 and 181 were analyzed on Western blot (Figure 6A). Juvenile HD fibroblast lysates showed a significant 10.1% (SEM ± 2.7%) lower level of mutant htt protein compared to wild-type htt (Figure 6B).

Next, we analyzed SDS-soluble levels of wild-type and mutant htt protein in post-mortem juvenile HD brain lysates. Three of the four juvenile HD brain lysates showed multiple mutant htt protein products (Figure 6C). Despite of the reduced quality of the post-mortem juvenile HD brain lysates, we were able to quantify the protein bands and showed 16.4% (SEM ± 7.0%) lower mutant htt protein levels with respect to wild-type htt (Figure 6D).

These results show that in adult-onset HD samples, wild-type and mutant htt protein levels are equal, regardless of mutant htt protein aggregation. In juvenile HD there is a consistent lower level of mutant htt protein expression.

### HTT antisense expression in HD patient-derived fibroblasts and post-mortem brain tissue

**Figure 7:**
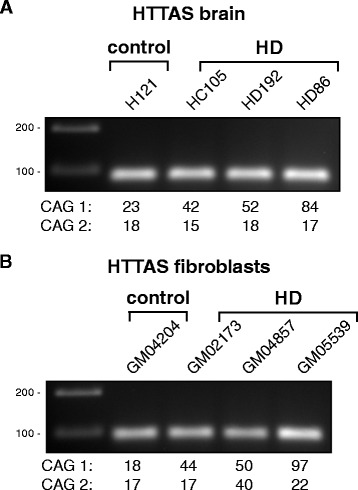
HTT antisense expression in HD patient-derived fibroblasts and brain tissue. Gel electrophoresis of HTTAS amplified using strand- and HTTAS isoform-specific primers. **(A)** RT-PCR of patient-derived fibroblasts from a control (GM04204), an HD patient (GM02173), an HD patient homozygous for the CAG repeat expansion (GM04857) and a juvenile HD patient (GM05539). **(B)** RT-PCR of post-mortem brain tissue from a control (H121), an HD patient (HC105), and 2 juvenile HD patients (HD192 and HD86). Allelic CAG repeat sizes below each lane.

It is known that a lower expression of HTTAS that contains the CTG repeat can increase HTT mRNA expression [8]. To investigate if the lower mutant HTT mRNA levels we observed were due to higher HTTAS expression we investigated HTTAS expression in fibroblasts and post-mortem brain tissue from HD patients. In post-mortem brain tissue we detected comparable levels of HTTAS in HD and control brain tissue (Figure 7A). We could also reliably detect HTTAS in all cell lines that we investigated, including the homozygous HD patient-derived fibroblasts (Figure 7B). Sanger sequencing confirmed that this was HTTAS with the expanded CTG repeat. This was unexpected since reduced expression of HTTAS with an expanded CTG repeat was reported in post-mortem HD brain [8], which would suggest no expression of this HTTAS when both *HTT* alleles contain an expanded repeat. We conclude that our observed variations in mutant and wild-type HTT mRNA levels in post-mortem brain are probably not caused by altered transcription of HTTAS with an expanded CTG repeat.

## Discussion

In the current study we found that in post-mortem adult-onset HD brain material the levels of wild-type and mutant HTT mRNA were significantly different. We found lower levels of mutant HTT mRNA compared to wild-type in the frontal cortex and a trend towards lower mutant HTT mRNA levels in the middle temporal gyrus. In adult-onset HD patient-derived fibroblasts the levels of wild-type and mutant HTT mRNA did not differ. This is in concordance with results found in patient-derived HD lymphoblasts, where it was shown that the expanded CAG repeat did not affect HTT mRNA expression [6]. Conversely, a small (~10%) upregulation of mutant HTT mRNA was recently shown in human post-mortem HD brain tissue using SNP-specific qPCR [9]. These contradictory results could be explained by the fact that the two SNP’s used in this study were both located outside HTT Exon 1 (3′UTR, and Exon 50). Furthermore, the small upregulation of mutant HTT mRNA was most pronounced in post-mortem brain material derived from patients with early neuropathological grades (grades 1 and 2), whereas the material in our study was generally of later disease stages (grades 3 and 4).

Wild-type and mutant htt protein levels did not significantly differ in either fibroblasts or post-mortem brain samples of adult-onset HD patients. Soluble htt has a half-life of approximately 24 hours [14] and we hypothesize that with Western blot analysis we detect soluble htt that is present in the cells. Protein aggregation is an important feature in HD brain tissue, but does not occur in HD fibroblasts [13], our results show that protein aggregation does not affect the levels of soluble htt protein. However, we did find lower mutant HTT mRNA levels in brain. A possible explanation could be an enhanced translation of mutant HTT mRNA, resulting in equal htt protein levels. Recently, increased translation of mutant HTT was suggested [7]. Cells overexpressing N-terminal htt fragments with a normal and mutant polyQ repeat showed an enhanced protein synthesis of htt fragments with an expanded polyQ repeat. This more efficient translation of mutant HTT mRNA was proposed to be caused by enhanced binding of the MID1-complex to the expanded CAG repeat and mediated by mTOR and S6K kinases [7].

Two of the three juvenile HD brain lysates showed multiple mutant htt protein products. This could be due to somatic mosaicism of mutant htt in the brain of juvenile patients [15] or due to reduced protein quality caused by the post-mortem delay. These multiple mutant htt protein products were not present in the fibroblast samples. Nonetheless, we consistently, found that the levels of mutant htt protein were lower than wild-type in both fibroblasts and post-mortem brain tissue of juvenile HD patients. This is in contradiction with previous studies in knock-in HD mice carrying one or two alleles with 111 CAG repeats [7], which showed increased mutant htt protein levels. The lower mutant htt protein level in juvenile HD is consistent with lower levels of mutant HTT mRNA. Although juvenile HD is much rarer than adult-onset HD [16], for development of a rapid phenotype HD animal models generally carry a mutant *HTT* transgene with juvenile CAG repeats [17]. Our results indicate that wild-type and mutant htt protein ratios are different in juvenile HD and adult-onset HD brain samples and this should be taken into account when interpreting results from HD models carrying a juvenile repeat expansion.

Recently it has been suggested that in polyQ disorders bidirectional RNA transcription could play a role in the disease pathology by deregulation of the sense transcript [8,18]. It was hypothesized that a bidirectional RNA transcript called HTTAS, negatively regulates htt transcript expression [8]. In accordance, in HD patient-derived fibroblasts and brains HTTAS with the expanded CTG repeat could not be detected [8]. Our results show HTTAS expression in all HD samples and most notably in patient-derived fibroblasts homozygous for the CAG repeat expansion, suggesting that there is an HTTAS with expanded CTG repeat transcribed. We can conclude however that differences in allelic HTT transcript levels in post-mortem brain are probably not caused by lower levels of HTTAS.

Recent advances have shown the potential of reducing mutant htt levels with oligonucleotide-based therapeutics. Reduction of both wild-type and mutant htt up to 70% was well tolerated in HD rodent models and non-human primates [19]. Long-term suppression of wild-type and mutant htt might not be desirable because of htt’s anti-apoptotic function [20] and importance for cell survival in the adult brain [21,22]. A different approach would be an allele-specific reduction of mutant htt. This could be achieved with oligonucleotides directed against SNPs unique to the mutant htt transcript, or by targeting the expanded CAG repeat directly [23]. Although our allelic HTT mRNA expression levels are in disagreement to that of Liu *et al.* [9], the overall differences in basal HTT mRNA in both studies are small. This shows that the levels of wild-type and mutant htt protein are not considerably different and provides feasibility for oligonucleotide therapeutics that are not completely specific for the mutant *HTT* allele.

## Conclusions

Although we found significantly lower mutant HTT mRNA levels in adult-onset HD, wild-type and mutant htt protein levels did not differ significantly in adult-onset HD brain samples. Conversely, in juvenile HD brain samples mutant htt protein levels were lower compared with wild-type htt, showing subtle differences between juvenile HD and adult-onset HD. Since most HD model systems harbor juvenile repeat expansions, our results suggest caution with the interpretation of htt mRNA and protein studies using HD cell and animal models with such long repeats. Furthermore, our HTTAS results in homozygous HD cells do not support reduced HTTAS expression due to an expanded CAG repeat.

## Methods

### Patient-derived fibroblasts and human brain samples

Fibroblasts derived from HD patients and controls were purchased from Coriell Cell Repositories, Camden, USA (Table 1). Fibroblasts were cultured at 37°C and 5% CO_2_ in Minimal Essential Medium (Gibco Invitrogen, Carlsbad, USA) with 15% heat inactivated Fetal Bovine Serum (Clontech, Palo Alto USA), 1% Glutamax (Gibco) and 100 U/ml penicillin/streptomycin (Gibco).

**Table 1:** **Patient-derived fibroblasts**

**Name**	**CAG 1**	**CAG 2**	**Type**	**Age at sampling**	**Age of onset**	**Sex**
GM00305	42	15	HD	56	46	F
GM02173	44	17	HD	52	NA	F
GM04022	44	18	HD	28	NA	F
GM04855	48	20	HD	11	26	M
GM04857	50	40	Homozygous HD	23	28	F
GM04281	71	17	Juvenile HD	20	14	F
GM05539	97	22	Juvenile HD	10	2	M
GM09197	179	18	Juvenile HD	6	NA	M
GM04204	18	17	Control	81	NA	M

M: male, F: female, NA: not assessed.

Samples ranked on CAG repeat size of the longest allele.

Post-mortem human brain tissue was obtained from the Neurological Foundation of New Zealand Human Brain Bank in the Centre for Brain Research, University of Auckland, and the brain bank from the department of Neurology, Leiden University Medical Center. Tissue was obtained with the families full consent and with the ethical approval of the various institutional Ethics Committees. For a complete list of samples and corresponding clinical information, see Table 2.

**Table 2:** **Post-mortem human brain tissue**

**Name**	**CAG 1**	**CAG 2**	**Type**	**Tissue**	**PMD**	**Grade**	**Age of death**	**Age of onset**	**Sex**
HC103	40	19	HD	MTG	11	1	41	35	M
HC105	42	15	HD	MTG	9	1	67	47	F
HD166	42	17	HD	FC	32	2	80	>70	M
HC102	43	17	HD	MTG	10	3	64	40	M
HC107	43	19	HD	MTG	3	3	75	58	M
HD193	44	9	HD	FC	18	3	68	44	M
HD188	44	16	HD	FC	NA	3	64	44	M
HD195	44	22	HD	FC	8.5	3	61	NA	F
HD159	47	17	HD	FC	42	3	41	26	F
HC114	47	21	HD	MTG	12	NA	53	30	F
HD192	52	18	Juvenile HD	FC	62	4	37	NA	M
HC104	53	18	Juvenile HD	MTG	15	3	40	15	M
HD86	84	17	Juvenile HD	FC	20	3	20	16	F
HD29	84	20	Juvenile HD	FC	11	NA	11	8	F
H121	23	18	Control	MTG	6	control	64	control	F

MTG: middle temporal gyrus, FC: frontal cortex, PMD: post-mortem delay (hours), Grade: neuropathological classification [28], M: male, F: female, NA: not assessed.

Samples ranked on CAG repeat size of the longest allele.

### CAG repeat sizing

Genomic DNA samples were isolated from patient-derived fibroblasts and human brain using the Wizard Genomic DNA Purification Kit (Promega, Madison, USA) according to manufacturer’s instructions and diluted to 50 μg/ml. The number of CAG repeats in the *HTT* gene was determined by PCR using primers “HD-1” and “HD-3” as described previously [24], followed by fragment analysis on an ABI 3130 Automated Capillary DNA Sequencer (Applied Biosystems, Life Technologies Corporation, Carlsbad, USA). The exact PCR conditions are available on request. The 3′ CAA and following CAG are not counted. For the polyQ repeat the CAA and CAG triplet are counted and the polyQ repeat is therefore 2 units longer than the CAG repeat size.

### RNA and genomic DNA analysis

Post-mortem brain tissue was homogenized using ceramic MagNA Lyser beads (Roche, Mannheim, Germany) by grinding in a Bullet Blender (Next Advance, Averill Park, USA) according to manufacturer’s instructions. Total RNA was isolated from fibroblast cells and brain tissue using the Aurum Total RNA Mini Kit (BioRad, Hercules, USA), with an on-column DNase treatment for 30 min. RNA was eluted in 40 μl elution buffer and cDNA was synthesized from 1 μg total RNA using the Transcriptor First Strand cDNA Synthesis Kit with oligo (dT) primers at 55°C for 90 min (Roche).

PCR was performed using 1 μl cDNA or genomic DNA, 10x Expand High Fidelity buffer with 15 mM MgCl_2_ (Roche), 200 μM dNTPs (Roche), 1 M Betaine (Sigma-Aldrich, St. Louis, USA), 15 pmol of both forward primer HttCAGFw: 5′-ATG GCG ACC CTG GAA AAG CTG AT-3′ and reverse primer HttCAGRev: 5′-TGA GGC AGC AGC GGC TG-3′ (Eurogentec, Liege, Belgium), 3 U Expand High Fidelity enzyme mix (Roche), and PCR grade water to a final volume of 30 μl. The PCR program started with a 2 min initial denaturation at 94°C, followed by 35 cycles of 15 sec denaturation at 94°C, 30 sec annealing at 60°C, 1 min elongation at 72°C, and final elongation step at 72°C for 7 min.

PCR products were loaded on a 2% agarose gel diluted in Tris/Borate/EDTA (TBE) buffer. DNA gel electrophoresis was performed for 1 hour at 100 V. Intensities of DNA bands were quantified using ImageJ software. Intensity of the HTT mRNA band was divided by the corresponding genomic DNA band to normalize for differences in PCR efficiency due to CAG repeat length.

### SNP genotyping and SNP linkage by circularization (SLiC)

The procedure for SNPs rs362273 genotyping and SNP linkage by circularization on human brain tissue was adapted from Liu *et al*. [11]. One μg of DNase-treated total RNA, together with oligo (dT) primers, was used for cDNA synthesis using SuperScript III First-Strand Synthesis System (Invitrogen). To improve reverse transcription of long cDNA templates, 2 M betaine and 0.6 M trehalose (both Sigma-Aldrich) were added to the reaction mixture [25]. cDNA synthesis was performed at 42°C for 2.5 hours, followed by RNase H treatment at 37°C for 20 min. Next, 5 μl cDNA was used as template for long-range PCR and SLiC.

### Taqman SNP assay

Quantitative PCR was performed using the LightCycler 480 II (Roche), according to manufacturer’s instructions, using a mixture containing 45 ng cDNA, 1xTaqMan Universal PCR Master Mix, no AmpErase UNG (Applied Biosystems), 1xTaqMan SNP Genotyping Assay (Applied Biosystems), and nuclease-free water (Ambion) in a 20 μl reaction volume. ACTB (Applied Biosystems, cat#Hs99999903_m1) was included as reference gene. A standard curve was generated using pooled equal amounts of cDNA from all samples. Quantification of the dual-color hydrolysis of both allele-specific fluorescent reporter dyes, FAM (“G” allele) and VIC (“A” allele), was performed with the LightCycler 480 SW 1.5.1 software (Roche) using the 2^nd^ derivative method, according to manufacturer’s instructions.

### HTT antisense determination

RNA isolation as described above. PCR was performed using 1.5 μl cDNA, 10x PCR buffer with 20 mM MgCl_2_ (Roche), 200 μM dNTPs (Roche), 6 pmol HTTAS_v1 primer, forward: 5′-CAC CGG GGC AAT GAA TGG-3′, reverse: 5′-GTG CGG ATG GCA AGG ACA G-3′, 2 U FastStart Taq DNA Polymerase (Roche), 1 M ethylene glycol (Sigma-Aldrich), and PCR grade water to a final volume of 30 μl. The PCR program started with a 3 min initial denaturation at 95°C, followed by 40 cycles of 10 sec denaturation at 95°C, 10 sec annealing at 60°C, 10 sec elongation at 72°C, after which a final elongation step was performed at 72°C for 7 min.

PCR products were loaded on a 3% TBE agarose gel and bands were extracted using the NucleoSpin Gel & PCR Clean-up kit (Machery Nagel, Düren, Germany). To confirm the sequence of HTTAS, PCR products were cloned into a pGEM-T Easy vector (Promega) and analyzed by Sanger sequencing using a T7-specific forward primer.

### Protein isolation

Fibroblasts were detached from the culture surface with a 0.5% Trypsin/EDTA solution. After washing twice with HBSS, cells were resuspended in 200 μl ice cold lysis buffer, containing 50 mM HEPES, 50 mM NaCl, 10 mM EDTA, 10 mM DTT, 0.1% CHAPS, and 1 tablet Complete mini protease inhibitor EDTA free (Roche) per 10 ml buffer [26]. Next, samples were sonicated 3 times 5 sec using ultrasound with amplitude 60 at 4°C. After 1 hour head-over-head incubation at 4°C, extracts were centrifuged for 15 min at 10,000 × *g* and 4°C and supernatant was isolated.

For brain homogenates, slices from frozen unfixed human brain tissue were collected using a sliding microtome (Leica SM 2010R). Tissue was homogenized using ceramic MagNA Lyser beads (Roche) by grinding in a Bullet Blender (Next Advance) for 3 min at strength 8 in lysis buffer with pH 7.2, containing 1% CHAPS, 137 mM NaCl, 2.7 mM KCl, 4.3 mM Na2PO4, 1.4 mM KH2PO4, 5 mM EDTA, 5 mM EGTA, and 1 tablet Complete mini protease inhibitor EDTA free (Roche) per 10 ml buffer [27]. Homogenates were incubated for 1 hour in a head-over-head rotator at 4°C, and centrifuged for 15 min at 10,000 × *g* at 4°C.

Protein concentrations were determined with the bicinchoninic acid kit (BCA) (Thermo Fisher Scientific, Waltham, USA) using Bovine Serum Albumin (BSA) as a standard. After addition of 5% glycerol, samples were aliquotted, snap frozen and stored at −80°C.

### Western blotting

SDS-PAGE separation of proteins was performed according to the “shorter CAG repeats” protocol as described previously [27]. Proteins were transferred to a 0.2 μm nitrocellulose membrane (Bio-Rad, #170-4159) using the Trans-blot Turbo (BioRad) at 2.5A (constant)/25 V for 10 min. Membranes were blocked for 15 min in tris buffered saline containing 5% non-fat milk (Nutricia, Schiphol, the Netherlands). Next, membranes were incubated with primary rabbit antibody EPR5526 (Abcam, Cambridge, UK) that recognizes the N-terminus of the htt protein, diluted 1:5000 in blocking buffer, followed by secondary incubation with rabbit IRDye800 (LI-COR, Lincoln, USA) diluted 1:5000 in blocking buffer. Blots were analyzed on an Odyssey reader (LI-COR). Protein bands corresponding to were quantified using the Odyssey software version 3.0 (LI-COR). Background correction was performed by sampling an empty area of the blot of the same size as the area that contained the positive protein band. Quantification of wild-type and mutant htt protein relied on densitometry measurement of both western blot bands separately. In case separation was small, we could magnify our scanned blots using the software’s “zoom” function to aid in a proper alignment of the densitometry calculation boxes over both separate bands. Wild-type and mutant htt protein expression levels relative to total htt protein expression were calculated by dividing wild-type and mutant htt band intensities with total htt band intensity (wild type + mutant).

### Statistical analyses

Experiments were performed at least six times per subject (3 biological and 2 technical replicates). PCR linearity was evaluated by GraphPad Prism version 6.02 by determining the individual linear regression coefficients (*r*2) of the band intensities of wild-type and mutant HTT expression versus the number of PCR cycles.

GraphPad Prism version 6.02 was used to create whisker boxplots (whiskers = min to max), showing all mean values per sample. IBM SPSS Statistics Version 20.0.0 was used for statistical analysis. All datasets were tested for a Gaussian distribution by visual inspection after plotting the residuals. Significance of the pairwise differences was determined using a linear mixed model.
